# A phase I/II study of combined weekly systemic cisplatin and locoregional hyperthermia in patients with previously irradiated recurrent carcinoma of the uterine cervix

**DOI:** 10.1038/sj.bjc.6690533

**Published:** 1999-07

**Authors:** R de Wit, J van der Zee, M E L van der Burg, W H Kruit, A Logmans, G C van Rhoon, J Verweij

**Affiliations:** Departments of Medical Oncology and Radiotherapy, Division of Hyperthermia, Rotterdam Cancer Institute and University Hospital, PO Box 5201, Rotterdam, 3008, AE, The Netherlands; Department of Gynaecology, Rotterdam Cancer Institute, Rotterdam, The Netherlands

**Keywords:** cervical cancer, cisplatin chemotherapy, hyperthermia

## Abstract

We investigated the feasibility and the anti-tumour activity of weekly cisplatin and the simultaneous application of local hyperthermia in patients with a pelvic recurrence of cervical cancer in previously irradiated area. Dose levels of cisplatin 60 mg m^−2^, 70 mg m^−2^ and 80 mg m^−2^ were studied. Treatment objective of hyperthermia was the achievement of a tumour temperature of ≥ 42° for 60 min, during cisplatin administration. The protocol advised six weekly cycles of combined treatment. Nineteen patients, median age 47 years (range 26–71), were treated. A total of 89 cycles of combined treatment were administered. Even at the highest dose level of cisplatin, 80 mg m^−2^ weekly, no dose-limiting toxicity was observed. Leucocytopenia at scheduled retreatment resulted in 1 or 2 weeks postponement in five cases. Neurotoxicity and renal toxicity were mild or absent. Maximum tumour temperatures achieved ranged 39.7–43.6°C, mean 41.6 ± 0.7°C. All 19 patients were evaluable for response. One patient achieved a complete response that lasted 20 months, and nine patients achieved a partial response for a median duration of 6 months (range 4–50+ months), for an overall response rate of 53%. One patient subsequently underwent salvage surgery and currently remains free of disease at 4 years. We found that this combined hyperthermia- dose-intensive cisplatin regimen was well-tolerated. The true impact of the combination of cisplatin and locoregional hyperthermia can only be answered in a randomized study. Nonetheless, based on existing data on the poor efficacy of cisplatin in pelvic recurrent cervical cancer, we believe that the combined modality approach of weekly hyperthermia plus dose-intensive cisplatin is an attractive regimen, particularly if subsequent salvage surgery is available. © 1999 Cancer Research Campaign


*British Journal of Cancer* (1999) **80**(9), 1387–1391
© 1999 Cancer Research Campaign
Article no. bjoc.1999.0533

### A phase I/II study of combined weekly systemic cisplatin and locoregional hyperthermia in patients with previously irradiated recurrent carcinoma of the uterine cervix

**R de Wit[1], J van der Zee[2], MEL van der Burg[1], WH Kruit[1], A Logmans[3,*], GC van Rhoon[2] and J Verweij[1]**

Departments of [1]Medical Oncology and [2]Radiotherapy, Division of Hyperthermia, Rotterdam Cancer Institute and University Hospital, PO Box 5201, 3008 AE Rotterdam, The Netherlands; [3]Department of Gynaecology, Rotterdam Cancer Institute, Rotterdam, The Netherlands

**Summary** We investigated the feasibility and the anti-tumour activity of weekly cisplatin and the simultaneous application of local hyperthermia in patients with a pelvic recurrence of cervical cancer in previously irradiated area. Dose levels of cisplatin 60 mg $m^{-2}$, 70 mg $m^{-2}$ and 80 mg $m^{-2}$ were studied. Treatment objective of hyperthermia was the achievement of a tumour temperature of ≥ 42° for 60 min, during cisplatin administration. The protocol advised six weekly cycles of combined treatment. Nineteen patients, median age 47 years (range 26–71), were treated. A total of 89 cycles of combined treatment were administered. Even at the highest dose level of cisplatin, 80 mg $m^{-2}$ weekly, no dose-limiting toxicity was observed. Leucocytopenia at scheduled retreatment resulted in 1 or 2 weeks postponement in five cases. Neurotoxicity and renal toxicity were mild or absent. Maximum tumour temperatures achieved ranged 39.7–43.6°C, mean 41.6 ± 0.7°C. All 19 patients were evaluable for response. One patient achieved a complete response that lasted 20 months, and nine patients achieved a partial response for a median duration of 6 months (range 4–50+ months), for an overall response rate of 53%. One patient subsequently underwent salvage surgery and currently remains free of disease at 4 years. We found that this combined hyperthermia-dose-intensive cisplatin regimen was well-tolerated. The true impact of the combination of cisplatin and locoregional hyperthermia can only be answered in a randomized study. Nonetheless, based on existing data on the poor efficacy of cisplatin in pelvic recurrent cervical cancer, we believe that the combined modality approach of weekly hyperthermia plus dose-intensive cisplatin is an attractive regimen, particularly if subsequent salvage surgery is available.

**Keywords:** cervical cancer; cisplatin chemotherapy; hyperthermia

Cisplatin is the most active single agent in cervical cancer, yielding a 21–31% response rate. However, in patients with a pelvic recurrence within previously irradiated areas the response rate is lower than in patients with extrapelvic sites of disease. In addition, responses in pelvic recurrences are usually partial at best, and of brief, median 4–6 months, duration. In in vitro and in vivo models marked synergism has been demonstrated of the simultaneous application of heat and cisplatin (Wallner et al, 1986, 1987; Baba et al, 1989). It appears that cytotoxic synergism is greatest when cells are exposed to cisplatin and hyperthermia simultaneously (Dahl, 1995). Synergism can already be demonstrated both in vitro and in vivo at 41 and 42°C, and there appears to be a linear increase in cisplatin cytotoxicity with increasing temperature (40–45°C). With the use of local deep hyperthermia, tumour temperatures of about 42–45°C can be reached and tolerated for 30–60 min. In addition, with the use of local hyperthermia and the systemic administration of chemotherapy, maximum synergism can be achieved, without increasing systemic side-effects, particularly on bone marrow and kidneys (Dahl, 1995). Therefore, the approach of combining local hyperthermia with chemotherapy provides a means of targeting and selective toxicity, thereby increasing cell kill in the tumour. In the present study, we investigated the maximum tolerated dose and the potential of the combination of weekly systemic administration of cisplatin with simultaneous application of local hyperthermia to the pelvic area induced by electromagnetic radiation. The chemotherapy regimen was based on our previous experience with weekly administration of cisplatin (Planting et al, 1993, 1997).

*Received 23 July 1998*
*Revised 6 January 1999*
*Accepted 21 January 1999*

*Correspondence to:* R de Wit

### PATIENTS AND METHODS

### Patients

Eligibility criteria required histologically proven pelvic recurrence of cervical cancer in previously irradiated area, not amenable to surgery. Patients had to have a lesion measurable in one or two dimensions within the field of combined treatment. For the purpose of measuring response, computerized tomography (CT) scanning was mandatory in all patients. Both squamous cell carcinoma and adenocarcinoma were eligible. Simultaneous metastatic disease outside the pelvis was not an exclusion criterion. Other eligibility criteria were performance status (WHO Scale) 0–2, normal bone marrow functions (white blood cells (WBC) above $3.5 \times 10^9$ $l^{-1}$ and platelets above $100 \times 10^9$ $l^{-1}$), serum creatinine below 120 μmol $l^{-1}$, or measured creatinine clearance above 50 ml $min^{-1}$. Patients with

*Present address: Zuiderziekenhuis, Groene Hilledijk 315, 3075 EA Rotterdam, The Netherlands.

prior systemic chemotherapy for recurrent tumour were excluded. A pacemaker or an artificial hip were absolute contraindications for hyperthermia treatment. Institutional review board-approved informed consent was obtained from all patients before study entry.

### Cisplatin chemotherapy

Cisplatin was administered once weekly. The administration of the chemotherapy started with prehydration with 1 litre of normal saline in 4 h. Cisplatin was then administered in 250 ml saline 3% over 3 h. An additional 2 litres of normal saline plus 40 mmol potassium chloride plus 4 g magnesium sulphate was infused over the next 16 h. As anti-emetic treatment, all patients received ondansetron 8 mg intravenous (i.v.) bolus plus dexamethasone 10 mg i.v. bolus before the start of cisplatin, followed by ondansetron 8 mg orally twice daily, and dexamethasone 6 mg once daily on days 2 and 3. If at scheduled retreatment WBC were below $2.5 \times 10^9\ l^{-1}$ and/or platelets were below $75 \times 10^9\ l^{-1}$, both the chemotherapy and hyperthermia treatment were postponed for 1 week. If the treatment had to be postponed for 3 weeks or more the patient went off study. If renal toxicity or neurotoxicity ≥ grade 2 was observed, treatment was stopped. Patients with evidence of progression at any time during treatment were taken off study and considered as progressive disease.

### Procedure of deep hyperthermia

Immediately after starting with the cisplatin infusion, installation for deep local hyperthermia of the pelvis commenced; including placement of thermometry probes, patient positioning, positioning of the applicator and surface and systemic cooling. For thermometry, probes were placed intraluminally in bladder, vagina and rectum within closed-tip catheters. Thermal mapping was performed every 5 min with a stepsize of 1 cm (van der Zee et al, 1998). Temperature measurements were distinguished between 'tumour contact', meaning that the site of measurement was in direct contact with tumour tissue, and 'tumour indicative', meaning that the site of measurements was within the heated volume around the tumour. Oral temperature was measured every 5 min. For hyperthermia, the BSD-2000 system was available, including Bowman probes for thermometry (Turner et al, 1989). Patients were lightly sedated with 1 mg lorazepam. Following preparations, heating was started with power output at 400 W. Patients were carefully instructed to mention any unpleasant sensation which might be the result of a hot spot, such as a burning sensation, a feeling of pressure, any pain, or bowel or bladder spasms. The treatment settings for frequency, amplitude distribution and phase shifting at the start of the first treatment were chosen on the basis of the two-dimensional pretreatment planning provided with the BSD-2000 system. Thereafter, treatment settings were adjusted depending on either information from E-field measurements or temperature distribution. Information on temperature distribution came from intraluminally placed thermometry probes, and from the patient. Any pain mentioned by the patient which disappeared within 1 min following power decrease was considered to indicate a too high temperature and the treatment settings were adjusted to decrease power input at the specific location. Adjustments of treatment settings could be either changes in power output per channel, frequency or phase settings, or placement of an additional water bolus. Power output was increased to as high as the patient could tolerate without pain.

**Table 1** Patient characteristics

| | |
|---|---|
| Age median (range) | 47 (26–71) |
| WHO performance | |
| 0 | 0 |
| 1 | 13 |
| 2 | 6 |
| Histology | |
| Squamous cell carcinoma | 12 |
| Adenocarcinoma | 5 |
| Mixed | 2 |
| Previous definitive radiotherapy | 10 |
| Previous surgery plus radiotherapy | 9 |
| Pelvic relapse | 17 |
| Pelvic relapse plus distant metastases | 2 |

Treatment objective was the achievement of a tumour T of ≥ 42°C for a period of 60 min.

The heating-up time was maximum 30 min, therefore effective heating was to take place during the second hour of the cisplatin administration. If during heating-up a temperature of 42°C could not be achieved within 30 min, the 60-min application started at that time.

### Criteria for response and toxicity evaluation and dose-escalation

The treatment schedule consisted of six weekly combined administrations of hyperthermia and cisplatin infusions. Response evaluation took place 4 weeks after the last treatment. For response evaluation and toxicity grading, with the exception of nausea and vomiting, the WHO criteria were used (WHO, 1979). Toxicity was reported as the worst grade observed during the whole treatment period. For grading of nausea and vomiting a modified grading system was used: grade 0: none; grade 1: mild to moderate nausea not interfering with adequate fluid and food intake; grade 2: nausea interfering with adequate fluid and or food intake and/or vomiting < 5 × in 24 h; grade 3: any nausea or vomiting worse than grade 2 but not requiring i.v. support; grade 4: any nausea and or vomiting for which hospital admission was necessary.

Patients were evaluable for response if they had completed three combined treatments, unless there was rapid early progression. Patients were evaluable for toxicity if they had received at least one combined treatment.

The starting dose of cisplatin was 60 mg $m^{-2}$, dose level 1. Dose level 2 consisted of cisplatin at a dose of 70 mg $m^{-2}$, dose level 3 of 80 mg $m^{-2}$. Since we had previously determined cisplatin 85 mg $m^{-2}$ $week^{-1}$ to be the maximum tolerable dose without hyperthermia (Planting et al, 1993), it was decided not to further escalate above 80 mg $m^{-2}$ in the present study. At least three patients were to be entered onto each dose level, until dose-limiting toxicity was observed. No intra-patient dose escalation was performed. If one instance of dose-limiting non-haematological and/or haematological toxicity were observed among three patients, an additional three patients were treated at the same dose level. If dose-limiting toxicity was observed, in only one or two of six patients, dose escalation was to be continued. If three instances of dose-limiting toxicity were observed among six patients, an additional three patients were to be treated at the preceding dose level. If dose-limiting toxicity was observed at this dose level, in only one or two patients, this dose level was declared the maximum tolerated dose (MTD). Dose limiting toxicity was

*© 1999 Cancer Research Campaign*

**Table 2** Cisplatin dose-intensity achieved

| Dose level | Cisplatin dose (per m²) | No patients/ no administrations | Mean total dose of cisplatin delivered (per m²) | Mean achieved dose intensity cisplatin (mg m$^{-2}$ week$^{-1}$) | Percentage delivered[a] |
|---|---|---|---|---|---|
| 1 | 60 | 5/23 | 276 | 53 | 96% |
| 2 | 70 | 9/40 | 311 | 59 | 95% |
| 3 | 80 | 5/25 | 400 | 72 | 89% |

[a]Denotes mean percentage cisplatin delivered of the planned dose, corrected for reason of withdrawal due to tumour progression.

**Table 3** Worst toxicity observed per patient

| Cisplatin dose (m²) | Patients (*n*) | Toxicity (WHO criteria) | | | | | | | | | | | | | | | | | | | |
|---|---|---|---|---|---|---|---|---|---|---|---|---|---|---|---|---|---|---|---|---|---|
| | | Myelotoxicity | | | | | Nausea/vomiting[b] | | | | | Neurotoxicity | | | | | Renal toxicity | | | | |
| | | 0 | 1 | 2 | 3 | 4 | 0 | 1 | 2 | 3 | 4 | 0 | 1 | 2 | 3 | 4 | 0 | 1 | 2 | 3 | 4 |
| 60 | 5 | 2 | 0 | 2 | 1[a] | 0 | 0 | 2 | 3 | 0 | 0 | 4 | 1 | 0 | 0 | 0 | 3 | 1 | 1 | 0 | 0 |
| 70 | 9 | 4 | 1 | 1 | 2[a] | 1[a] | 1 | 5 | 2 | 1 | 0 | 8 | 1 | 0 | 0 | 0 | 7 | 2 | 0 | 0 | 0 |
| 80 | 5 | 1 | 1 | 1 | 2[a] | 0 | 1 | 1 | 1 | 2 | 0 | 1 | 3 | 1 | 0 | 0 | 4 | 1 | 0 | 0 | 0 |

[a] Denotes leucocytopenia (four cases) and thrombocytopenia (one case), causing 1–2 weeks delay in retreatment. [b]Denotes nausea/vomiting, using modified criteria, as depicted in Patients and Methods.

defined as thrombocytopenia grade 4, or neutropenia grade 3 or 4 with fever, renal toxicity ≥ grade 2, neurotoxicity ≥ grade 2, any other non-haematological toxicity grade ≥ 3.

The dose intensity of cisplatin was calculated as the total amount of cisplatin administered divided by the total number of treatment weeks necessary to administer the total dose and is expressed in milligrams per square meter per week: in patients completing six treatment cycles in 6 weeks the total dose is divided by 6: in case of treatment delay the total dose administered is divided by 6 + the number of weeks delay.

### RESULTS

A total of 19 patients were treated. Patient characteristics are shown in Table 1. Seventeen patients had pelvic recurrence within previously irradiated area as the sole site of disease. Two patients simultaneously had distant metastases; one had liver metastases, the other had pulmonary metastases. One patient had previously been treated with bleomycin, vindesine, mitomycin-c, cisplatin (BEMP) induction chemotherapy before definitive radiotherapy.

A total of 89 cycles of local hyperthermia and systemic cisplatin were administered, the numbers of cycles at each dose-level, the mean total dose of cisplatin and dose-intensity achieved are shown in Table 2. Since several patients at dose-level 2 (70 mg m$^{-2}$) stopped treatment after three cycles for non-treatment-related reasons (see below), additional patients were entered onto dose-level 2 to increase the number of patients that received more than three cycles. One cycle of cisplatin in one patient was administered without hyperthermia due to system failure. Leucocytopenia grade 3 (five cases) necessitated postponement of retreatment at cycle 5 or 6 for 1 or 2 weeks, and resulted in 10–15% reduced dose-intensities in the three dose-levels. The median number of cycles administered per patient was five (range 3–6). The reasons to stop treatment before the six scheduled cycles were progressive disease (five patients with early progression after three cycles), toxicity (two patients after three, four and five cycles each), and refusal (one patient after five cycles).

The median tumour volume treated was 150 cm$^3$ (range 21–425) cm$^3$. Power was applied to a maximum varying from 300 to 900 W with an average of 607 W (median 600). Maximum tumour contact temperatures achieved ranged from 39.7–43.6°C with a mean of 41.6 ± 0.7°C (median 41.6). Maximum tumour indicative temperatures ranged from 39.3–43.7°C with a mean of 41.8 ± 0.7°C (median 42.1). The oral temperature increased with a mean of 1.2°C (median 1.1) to a maximum value of 37.2–38.9°C.

### Toxicity

The worst toxicity observed in each patient is shown in Table 3. Even at the highest dose of cisplatin 80 mg m$^{-2}$ week$^{-1}$ no dose-limiting toxicity was observed. As indicated above, grade 3 leucocytopenia at scheduled retreatment resulted in 1 or 2 weeks delay in five cases. One patient had grade 4 thrombocytopenia after four cycles and simultaneously refused further treatment, because a vesicovaginal fistula had developed. This patient had previously been treated with BEMP induction chemotherapy. Nausea and vomiting, predominantly occurring during days 2–4 after the chemotherapy (delayed emesis) developed and worsened during subsequent courses of chemotherapy and resulted in cessation of treatment in two patients after five cycles. Neurotoxicity was mild; grade 1 (five patients) and grade 2 (tinnitus in one patient), and did not result in withdrawal from protocol treatment. Renal toxicity grade 1 (four patients) and grade 2 (one patient) was related to renal function impairment (measured creatinin clearance 50–60 ml min$^{-1}$) at the start of treatment and did not necessitate treatment cessation.

The combined treatment was generally well-tolerated. Hyperthermia was delivered during 90 min during all cycles in 14 patients; in five patients 11 treatments were stopped after 66–86 min due to intolerable discomfort. In two patients a subcutaneous

*© 1999 Cancer Research Campaign*

burn located in the upper leg resulted as a direct hyperthermia induced toxicity. The clinical symptoms were limited to an induration in the subcutaneous fat which was tender for 2–3 days and gradually disappeared.

### Responses

All 19 patients were evaluable for response. One patient at dose level 3 (80 mg $m^{-2}$) achieved a complete response (CR) that lasted 20 months. Upon relapse of the pelvic tumour this patient was retreated with six weekly cisplatin cycles plus hyperthermia, and again achieved a near CR. This second response is now lasting for 9+ months. In addition, nine patients achieved a partial response (PR); two out of five patients at dose level 1 (60 mg $m^{-2}$, five out of nine patients at dose level 2 (70 mg $m^{-2}$) and two additional patients (out of five) at dose level 3. The median duration of these PRs is 6 months (range 4–54+ months). The patient with 54+ months progression-free survival obtained a near CR upon completion of the protocol treatment, and subsequently underwent pelvic exenteration salvage surgery. Histological examination revealed microscopic foci of persistent viable tumour. Therefore, ten out of 19 patients obtained a response, for an overall response rate of 53%. Of the remaining patients, one had a NC in the pelvic tumour after three cycles, but stopped treatment because she had progressive liver metastases. One patient had NC after six cycles that lasted five months, and seven patients had progression (PD) at the time of evaluation. There were no differences between responders and non-responders for tumour contact temperatures, tumour indicative temperatures, tumour volume, oral temperature increase or total power applied (data not shown).

### DISCUSSION

Surgery or radiotherapy, or a combination of these two modalities, continue to be the primary treatment options for invasive cervical cancer. However, following surgery plus post-operative radiotherapy, or primary definitive radiation therapy, or radiotherapy for recurrent pelvic disease up to 40% of patients will develop pelvic recurrent disease. With the exception of salvage surgery as a treatment option in some of these patients, the use of systemic chemotherapy is the only remaining treatment modality. Cisplatin has emerged as the most active single agent for treating patients with metastatic disease; no other standard cytotoxic drug has been associated consistently with objective response rates of 25% or higher. However, in patients who experience relapses following definitive radiation therapy cisplatin has only a minor effect, if any, on the natural history of the disease (Brader et al, 1998). The effectiveness of chemotherapy for patients with recurrent cervical cancer is compromised by the problems of drug distribution resulting from prior pelvic irradiation (Hopewell, 1983). In addition, it is likely that recurrent or persistent foci of cancer after radiotherapy represent more resistant disease (Osmak et al, 1989).

In a study by Potter et al (1989) the complete response rate to cisplatin in patients with distant metastases was 53%, with an overall response rate of 73%, whereas no complete responses and no more than seven partial responses (21%) were obtained in 33 patients with localized pelvic recurrence or persistent disease (Potter et al, 1989). In a report by Lele et al (1989) on 67 patients with cervical cancer who were treated with weekly cisplatin chemotherapy, the response rates by site were: liver 33%, lymph nodes 40%, lung 48%, whereas only one out of 24 patients with a pelvic recurrence (4%) obtained a response. Therefore, new approaches to the management of pelvic recurrent disease are clearly warranted.

Research in animal and human cell cultures has provided evidence that a number of chemotherapeutic agents, cisplatin in particular, have cytotoxicity that is significantly enhanced at elevated temperatures. Cytotoxicity of cisplatin increases almost linear with increasing temperature and maximal potentiation occurs when hyperthermia and cisplatin are administered simultaneously. The exact mechanism of potentation remains to be elucidated, but increased intracellular uptake, as well as increased DNA damage in the interactive effect, and impairment of DNA strand-break repair have been shown (Dahl, 1995).

The application of deep local hyperthermia with the systemic administration of cisplatin in patients with pelvic recurrent cervical cancer thus appears an attractive notion. Following initial feasibility data on the clinical use of combined cisplatin and local hyperthermia treatment (Green et al, 1989), Rietbroek et al recently reported on a phase II study of combined weekly loco-regional hyperthermia and systemic administration of cisplatin in patients with previously irridiated recurrent cervical carcinoma (Rietbroek et al, 1996, 1997). By using a regimen of cisplatin of 50 mg $m^{-2}$ $week^{-1}$ with 1 week interruption after every four cycles for a total of 12 cycles, projected dose-intensity 40 mg $m^{-2}$ $week^{-1}$, these authors observed an overall response in 12 of 23 patients, 52% (95% confidence interval (CI) 31–73%). Additional salvage surgery became possible in three responding patients, whose tumours were previously considered unresectable.

We conducted a phase I/II study based on our previous experience with weekly cisplatin at a considerably higher dose-intensity (Planting et al, 1993, 1997); weekly local hyperthermia was combined with cisplatin for a total of six cycles at cisplatin dose levels (and projected dose-intensity of six cycles in 6 weeks) 60, 70 and 80 mg $m^{-2}$ $week^{-1}$. We found that this combined hyperthermia-dose-intensive cisplatin regimen was well-tolerated, with no dose-limiting toxicity observed at the highest dose level of 80 mg $m^{-2}$ $week^{-1}$ tested. Cisplatin was not escalated above 80 mg $m^{-2}$, since we had previously demonstrated in patients treated with cisplatin without concurrent hyperthermia that cisplatin 80 mg $m^{-2}$ weekly is the maximum tolerated dose. We have thus demonstrated that local hyperthermia and cisplatin can be safely combined and that hyperthermia does not adversely impact the tolerability of cisplatin given at maximum tolerated single modality dose.

With the use of this weekly times 6 hyperthermia plus dose-intensive cisplatin regimen we obtained one complete response and nine partial responses in a total of 19 patients, for an overall response rate of 53%. The median duration of response was 6 months (range 4–54+ months).

One patient with a near complete response subsequently underwent salvage surgery and currently remains free of disease at 4 years.

The true impact of the use of the combination of cisplatin and locoregional hyperthermia can only be answered in a randomized study of chemotherapy alone versus the combined treatment. Nonetheless, based on the existing data of the poor efficacy of cisplatin when used as single treatment modality, and the favourable reports on the combined treatment now available which resulted in over 50% response rates in previously irradiated pelvic recurrent cervical carcinoma (Rietbroek et al, 1997 and present study), we believe that the combined modality approach of weekly

 *© 1999 Cancer Research Campaign*

hyperthermia plus dose-intensive cisplatin is an attractive induction regimen, particularly in patients for whom the option of subsequent salvage surgery is available.

### REFERENCES

Baba H, Siddik ZH, Strebel FR, Jenkings GN and Bull JMC (1989) Increased therapeutic gain of combined cisdiamminedichloroplatinum (II) and whole body hyperthermia by optimal heat scheduling in the rat. *Cancer Res* **49**: 7041–7044

Brader KR, Morris M, Levenback C, Levy L, Lucas KR and Gershenson DM (1998) Chemotherapy for cervical carcinoma: factors determining response and implications for clinical trial design. *J Clin Oncol* **16**: 1879–1884

Dahl O (1995) Interaction of heat and drugs in vitro and in vivo. In: *Thermoradiotherapy and Thermochemotherapy*, vol. 1, Seegenschmiedt MH, Fessenden P and Vernon CC (eds), pp. 103–121. Springer: Berlin

Green DM, Burton GV, Cox EB, Hanson D, Moore J and Oleson JR (1989) A phase I/II study of combined cisplatin and hyperthermia treatment for refractory malignancy. *Int J Hyperthermia* **5**: 13–21

Hopewell JW (1983) Radiation effects on vascular tissue. In: *Cytotoxic Insult to Tissue: Effect on Cell Lineages*, Potten CS, Hendry JH (eds), pp. 228–257. Churchill Livingstone: Edinburgh

Lele SB and Piver MS (1989) Weekly cisplatin induction chemotherapy in the treatment of recurrent cervical carcinoma. *Gynecol Oncol* **33**: 6–8

Osmak M and Perovic S (1989) Multiple fractions of gamma rays induced resistance to cis-dichloro-diammineplatinum (II) and methotrexate in human HeLa cells. *Int J Radiat Oncol Biol Phys* **16**: 1537–1541

Planting AST, van der Burg MEL, de Boer-Dennert M, Stoter G and Verweij J (1993) Phase I/II study of a short course of weekly cisplatin in patients with advanced solid tumours. *Br J Cancer* **68**: 798–792

Planting AST, de Mulder PHM, de Graeff A and Verweij J (1997) Phase II study of weekly high-dose cisplatin for six cycles in patients with locally advanced squamous cell carcinoma of the head and neck. *Eur J Cancer* **33**: 61–65

Potter ME, Hatch KD, Potter MY, Shingleton HM and Baker VV (1989) Factors effecting the response of recurrent squamous cell carcinoma of the cervix to cisplatin. *Cancer* **63**: 1283–1286

Rietbroek RC, Bakker PJM, Schilthuis MS, Postma AJ, Zum Vörde Sive Vörding PJ, González González D, Kurth KH, Bakker AJ and Veenhof CHN (1996) Feasibility, toxicity and preliminary results of weekly locoregional hyperthermia and cisplatin in patients with previously irradiated recurrent cervical carcinoma or locally advanced bladder cancer. *Int J Radiat Oncol Biol Phys* **34**: 887–893

Rietbroek RC, Schilthuis MS, Bakker PJM, Dijk van JDP, Postma AJ, González González D, Bakker AJ, van der Velden J, Helmerhorst THM and Veenhof CHN (1998) Phase II trial of weekly locoregional hyperthermia and cisplatin in patients with a previously irradiated recurrent carcinoma of the uterine cervix. *Cancer* **79**: 935–943

Turner PF and Schaefermeyer T (1989) BSD-2000 approach for deep local and regional hyperthermia: clinical utility. *Strahlenther Onkol* **165**: 700–704

van der Zee J, Peer-Valstar JN, Rietveld PJM, de Graaf-Strukowska L and van Rhoon GC (1998) Practical limitations of interstitial thermometry during deep hyperthermia. *Int J Radiat Oncol Biol Phys* **40**: 1205–1212

Wallner KE, de Gregorio MW and Li GC (1986) Hyperthermic potentiation of cisdamminedichloroplatinum (II) cytotoxicity in Chinese hamster ovary cells resistant to the drug. *Cancer Res* **46**: 6242–6245

Wallner KE and Li GC (1987) Effect of drug exposure duration and sequencing on hyperthermic potentiation of mitomycin-C and cisplatin. *Cancer Res* **47**: 493–495

WHO (1979) Handbook of Reporting Results of Cancer Treatment. WHO: Geneva

*© 1999 Cancer Research Campaign*
